# Hexose Oxidase-Mediated Hydrogen Peroxide as a Mechanism for the Antibacterial Activity in the Red Seaweed *Ptilophora subcostata*

**DOI:** 10.1371/journal.pone.0149084

**Published:** 2016-02-11

**Authors:** Kimi Ogasawara, Kenji Yamada, Noriyuki Hatsugai, Chiaki Imada, Mikio Nishimura

**Affiliations:** 1 Department of Cell Biology, National Institute for Basic Biology, Okazaki 444–8585, Japan; 2 Graduate School of Marine Science and Technology, Tokyo University of Marine Science and Technology, 4-5-7 Konan, Minato-ku, Tokyo 108–8477, Japan; 3 Research Center for Cooperative Projects, Hokkaido University, Kita-ku, Sapporo 060–8638, Japan; Griffith University, AUSTRALIA

## Abstract

Marine algae have unique defense strategies against microbial infection. However, their mechanisms of immunity remain to be elucidated and little is known about the similarity of the immune systems of marine algae and terrestrial higher plants. Here, we suggest a possible mechanism underlying algal immunity, which involves hexose oxidase (HOX)-dependent production of hydrogen peroxide (H_2_O_2_). We examined crude extracts from five different red algal species for their ability to prevent bacterial growth. The extract from one of these algae, *Ptilophora subcostata*, was particularly active and prevented the growth of gram-positive and -negative bacteria, which was completely inhibited by treatment with catalase. The extract did not affect the growth of either a yeast or a filamentous fungus. We partially purified from *P*. *subcostata* an enzyme involved in its antibacterial activity, which shared 50% homology with the HOX of red seaweed *Chondrus crispus*. In-gel carbohydrate oxidase assays revealed that *P*. *subcostata* extract had the ability to produce H_2_O_2_ in a hexose-dependent manner and this activity was highest in the presence of galactose. In addition, *Bacillus subtilis* growth was strongly suppressed near *P*. *subcostata* algal fronds on GYP agar plates. These results suggest that HOX plays a role in *P*. *subcostata* resistance to bacterial attack by mediating H_2_O_2_ production in the marine environment.

## Introduction

Marine algae, like terrestrial higher plants, are plagued by diseases caused by pathogenic bacteria (reviewed by [1–4]). It has been suggested that marine algae may be susceptible to disease caused by opportunistic bacteria, that become pathogenic in response to environmental change or decrease in host defense [2]. Therefore, little is known about the molecular mechanisms underlying defense against pathogen attack in marine algae. In contrast, the immune systems of terrestrial plants have been well-documented (reviewed by [5–6]). Marine algae are known to share basic mechanisms for pathogen recognition and signaling with terrestrial plants (reviewed by [1, 3, 7]). For example, oxidized polyunsaturated fatty acids, collectively known as oxylipins, play regulatory roles in the immune responses of certain marine algae, as well as terrestrial plants [8–9]. Another example of the conserved immune response between marine algae and terrestrial plants is microbe-associated molecular patterns (MAMPs)-induced immune responses. In terrestrial higher plants, Flg22, a 22-amino acid peptide in the N-terminal part of flagellin, is known to induce an immune response [10–12]. In female gametophytes of the red alga, *Saccharina japonica*, the Flg22-induced immune response is associated with an oxidative burst, a rapid and massive production of reactive oxygen species (ROS), such as superoxide (O_2_^-^) and hydrogen peroxide (H_2_O_2_) [13]. In red algae, such as *Gracilaria conferta* and *Chondrus crispus*, cell-wall oligosaccharides trigger a signal transduction cascade leading to an oxidative burst and a hypersensitive response (HR), which is local, and rapid programmed cell death [5, 14–18]. In marine algae, pharmacological studies have suggested that oxidative bursts are induced by an NADPH oxidase; this mechanism is similar to the oxidative burst that occurs during the immune response in terrestrial higher plants [3, 7, 19, 20]. Additionally, *in silico* research into the genome of the brown alga, *Ectocarpus siliculosus*, identified a number of genes encoding proteins that contain ligand-binding and signal-transduction domains, such as leucine-rich or tetratricopeptide repeat (LRR/TPR) domains, which are involved in direct or indirect pathogen recognition in higher plants [21]. On the other hand, marine algae have evolved their own defense system. Indeed, many algae appear to constitutively produce defense compounds, such as halogenated secondary metabolites, at high levels [22–23]. For example, the red alga *Bonnemaisonia hamifera* is coated with the metabolites at sufficiently high concentrations to protect itself against bacteria [24].

Investigations of the immune responses of algae (seaweeds) at various biological levels are necessary to determine the impact of biotic interactions in the marine environment and to understand the evolution of innate immunity in eukaryotes. In this study, we investigated the antibacterial activity of extracts from five red algal species. We partially purified an enzyme involved in the antibacterial activity of the red alga *P*. *subcostata*; the enzyme was identified by homology analysis as a hexose oxidase (HOX). We propose that HOX-mediated H_2_O_2_ production represents a defense mechanism against a broad range of bacteria in marine algae.

## Materials and Methods

### Red algal material and preparation of crude extracts

The red algae *Ptilophora subcostata*, *Scinaia japonica*, *Galaxaura elegans*, *Callophyllis japonica*, and *Gelidium elegans* were collected in the intertidal during zone summer on the Enoshima shore (GPS coordinates 35°18'28.2"N, 139°29'13.9"E), Kanagawa, Japan. Collection did not require specific permission and these red algae are not protected species. Freshly collected fronds were washed with cold water and stored at −80°C. For each sample, 1.8 g of algal fronds were homogenized in 25 mM Tris-HCl buffer (pH 7.2) and centrifuged at 15,000 *g* for 15 min. The volume of the supernatant was adjusted to 50 ml with 25 mM Tris-HCl buffer (pH 7.2).

### Antibacterial activity assay

An agar well diffusion assay was performed as described previously [25]. Spores of *B*. *subtilis* ATCC 6633 (10^7^ CFU/ml) were spread onto GYP agar plates containing 2% (w/v) D-glucose (Wako), 0.5% (w/v) yeast extract (Difco), 0.5% (w/v) Bacto-peptone (Difco), and 1.5% (w/v) agar (Difco), pH 6.0. Holes were made in the agar plates using a 5-mm diameter cork borer. Then, 60 μl of algal extract sample were added to each hole. The agar plates were incubated at 4°C for 2 h followed by 37°C for 16 h. The antibacterial activity was estimated by measuring the diameter of each clear growth-inhibition zone.

Alternatively, to measure antibacterial activity, we used a 96-well microtiter plates assay methods, which we developed. A total of 196 μl of *B*. *subtilis* ATCC 6633 (10^5^ CFU/ml) culture and 4 μl of algal extract sample were added to each well, and the plate was cultured in GYP liquid medium containing 2% (w/v) D-glucose, 0.5% (w/v) yeast extract, and 0.5% (w/v) Bacto-peptone, pH 6.0 at 37°C for 16 h. The algal extract samples were serially diluted ten-fold and then applied to each well. After incubation, the optical density at 660 nm (OD 660) was measured using a microtiter plate reader (MTP-300, Corona). We converted the antibacterial activity of the *P*. *subcostata* extracts to kanamycin resistance values. *B*. *subtilis* ATCC 6633 was cultured with or without kanamycin in 96-well microtiter plates as discussed above and the OD 660 nm was measured (S1 Fig). We used the following criteria for antibacterial activity: an OD 660 nm of <0.2 indicated antibacterial activity and that activity value was converted to 0.025 μg of kanamycin activity, and an OD 660 nm > 0.2 indicated no antibacterial activity. The total activity was calculated from the dilution ratio and the kanamycin volume.

### Effect of catalase on antibacterial activity of *P*. *subcostata* extracts

The algal extracts were incubated with 0, 1, 2 mg/ml of catalase (E.C.1.11.1.6, Sigma) in 25 mM Tris-HCl buffer (pH 7.2) at 37°C for 16 h. The catalase-treated extracts of *P*. *subcostata* were applied on agar diffusion assay.

### Heat treatment and ultrafiltration

The extracts were treated at 30, 40, 50, 60, 70, 80, 90 or 100°C for 10 min. For ultrafiltration, three (10, 50, and 100 kDa) molecular-weight-cutoff filters (Millipore) were used. The samples were subjected to an agar well diffusion assay to measure antibacterial activity.

### Purification of antibacterial components from *P*. *subcostata*

For each sample, 274 g of algal fronds were homogenized with 25 mM Tris-HCl (pH 7.2) and centrifuged at 15,000 *g* for 15 min. Ammonium sulfate was added to the supernatant to yield a 100% saturated solution and proteins were precipitated by centrifugation. The pellets were resuspended in 25 mM Tris-HCl buffer (pH 7.2) and dialyzed overnight against 25 mM Tris-HCl buffer (pH 7.2) to remove salt. The dialyzed samples were subjected to anion exchange chromatography on DEAE Sepharose FF (250 ml, Amersham Biosciences) and the column was eluted with 0.17 M, 0.27 M, and 0.37 M NaCl in Tris-HCl buffer (pH 7.2). The 0.27 M NaCl fraction, which had antibacterial activity, was subjected to gel filtration chromatography using Toyopearl HW-65S resin (particle size 20–40 μm, Tosoh), and the column was eluted with 25 mM Tris-HCl buffer (pH 7.2) at a 24 ml/min flow rate.

### Hexose oxidase activity assay

HOX activity was measured by coupling with ferrous oxidation-xylenol orange (FOX assay) as described previously [26]. Samples were incubated with 2% (w/v) of each hexose compound (D-glucose, lactose, cellobiose, D-galactose, and maltose) as substrate in 20 μl of 25 mM Tris-HCl buffer, pH 7.2 at 37°C for 16 h. The amount of H_2_O_2_ was measured using the FOX assay.

### In-gel carbohydrate oxidase assay

The in-gel carbohydrate oxidase assay was performed as described previously [27]. Samples after gel filtration were separated on an 8% native-PAGE gel, which was then stained with 0.5 mg mL^-1^ 4-chloro-1-naphthol, 5 units mL^-1^ horseradish peroxidase, and 100 mM D-glucose in 25 mM Tris-HCl buffer, pH 7.2. Activity appeared as a blue band on a clear background. GOX from *Aspergillus niger* was used as the positive control.

### Determination of N-terminal amino acid sequence

Extracts from *P*. *subcostata* were subjected to SDS–PAGE and then transferred electrophoretically to a PVDH membrane (0.22 μm pore size; Millipore). After CBB staining, 29 kDa and 40 kDa polypeptides were excised from the membrane and subjected to automatic Edman degradation on a peptide sequencer (Model 492, Applied Biosystems).

## Results

### Broad-spectrum antibacterial activity of a *P*. *subcostata* extract

**Fig 1:**
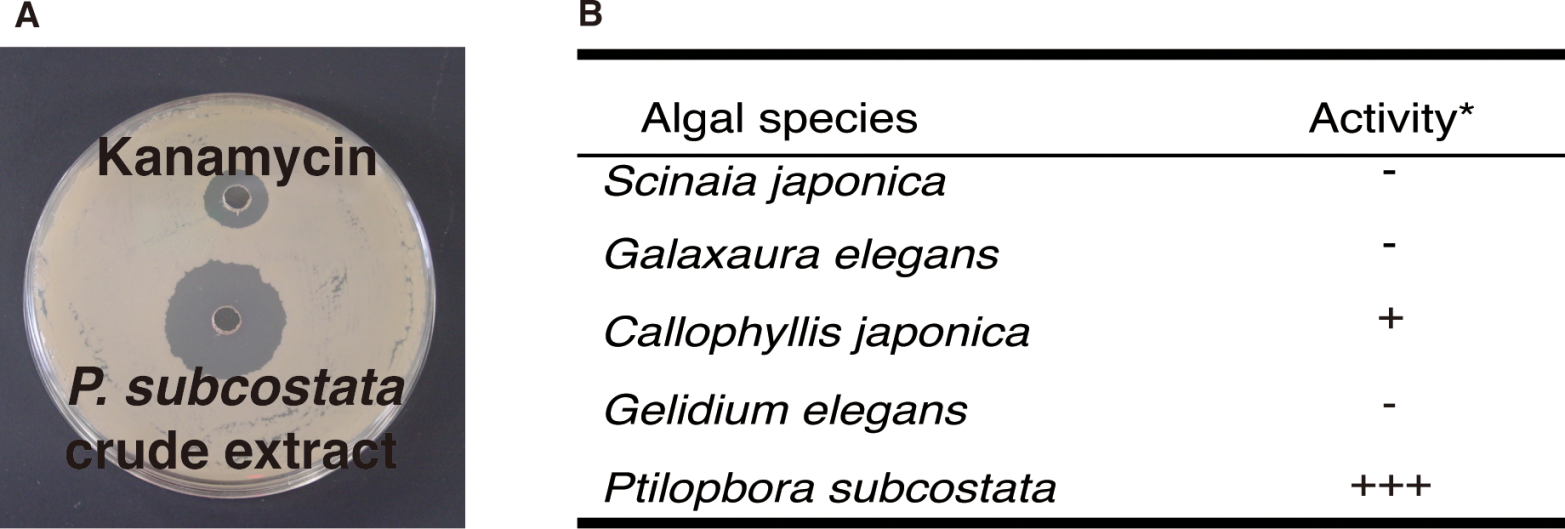
Antibacterial activity of extracts from five red algae. The algal extracts were applied to GYP agar plates onto which *B*. *subtilis* spores had been spread and their antibacterial activities were estimated by measuring the diameter of the clear inhibition zones after 16 h of incubation at 37°C. (A) Agar well diffusion assay showing inhibition of *B*. *subtilis* growth by *P*. *subcostata* extract. Upper, treated with kanamycin sulfate solution as a control. Lower, treated with *P*. *subcostata* extract. Growth-inhibition zones were observed. (B) Antibacterial activity of five red algae. *Diameter of growth-inhibition zone: -, 0 mm; +, ~7 mm; ++, 7–10 mm; +++, ≥10 mm.

To assay marine algae for antibacterial activity, we measured the effects of extracts from five red algal species—*S*. *japonica*, *Galaxaura elegans*, *C*. *japonica*, *Gelidium elegans*, and *P*. *subcostata*—on the growth of the gram-positive bacterium *B*. *subtilis* using an agar well diffusion assay. An agar plate spotted with extracts from *P*. *subcostata* displayed clear growth-inhibition zones of over 10 mm in diameter (Fig 1A). Extracts from *C*. *japonica* displayed growth-inhibition zones of less than 7 mm in diameter (Fig 1B). Extracts from *S*. *japonica*, *Galaxaura elegans*, and *Gelidium elegans* did not affect *B*. *subtilis* growth (Fig 1B). In addition, we examined the growth-inhibitory effects of extract from *P*. *subcostata* against gram-positive bacteria, gram-negative bacteria, a yeast, and a filamentous fungus. The algal extract prevented the growth of all of the bacteria tested (Table 1). In contrast, the yeast and filamentous fungus were not affected by the algal extract (Table 1). These results suggest that *P*. *subcostata* has activity against a variety of bacteria.

**Table 1:** Antibacterial spectrum of *P*. *subcostata* extract against bacterial strains, yeast and filamentous fungus.

Strain	Antibacterial activity*
*Escherichia coli k-12*	+++
*Escherichia coli JCM 1649*^*T*^	+++
*Pseudomonas aeruginosa IAM 1415*^*T*^	+++
*Staphylococcus aureus IAM 1098*	+++
*Bacillus subtilis ATCC 6633*	+++
*Bacillus subtilis ATCC 6633 spore*	+++
*Bacillus subtilis NRIC 0068*	+++
*Bacillus subtilis NRPL B-558*	+++
*Bacillus subtilis PCI 219*	+++
*Bacillus cereus IAM 1729*	+++
*Bacillus megaterium NRIC 1009*	+++
*Listeria monocytogenes IDD 577*	+++
*Listeria monocytogenes IDD 578*	+++
*Listeria monocytogenes IDD 579*	+++
*Listeria monocytogenes IDD 580*	+++
*Candida albicans 3147*	-
*Penicillium decambens IAM 7275*	-

* Diameter of growth-inhibition zone; -, 0 mm; +, ~7 mm; ++, 7~10 mm; +++, 10 mm~.

### Characterization of antibacterial activity in *P*. *subcostata* extract

We examined the pH sensitivity of the antibacterial activity of the *P*. *subcostata* extract. The antibacterial activity of the extract was highest in Tris-HCl buffer, pH 7.2 and activity decreased with increasing pH. Therefore, we used Tris-HCl buffer, pH 7.2 for extraction of the antibacterial substance(s).

**Fig 2:**
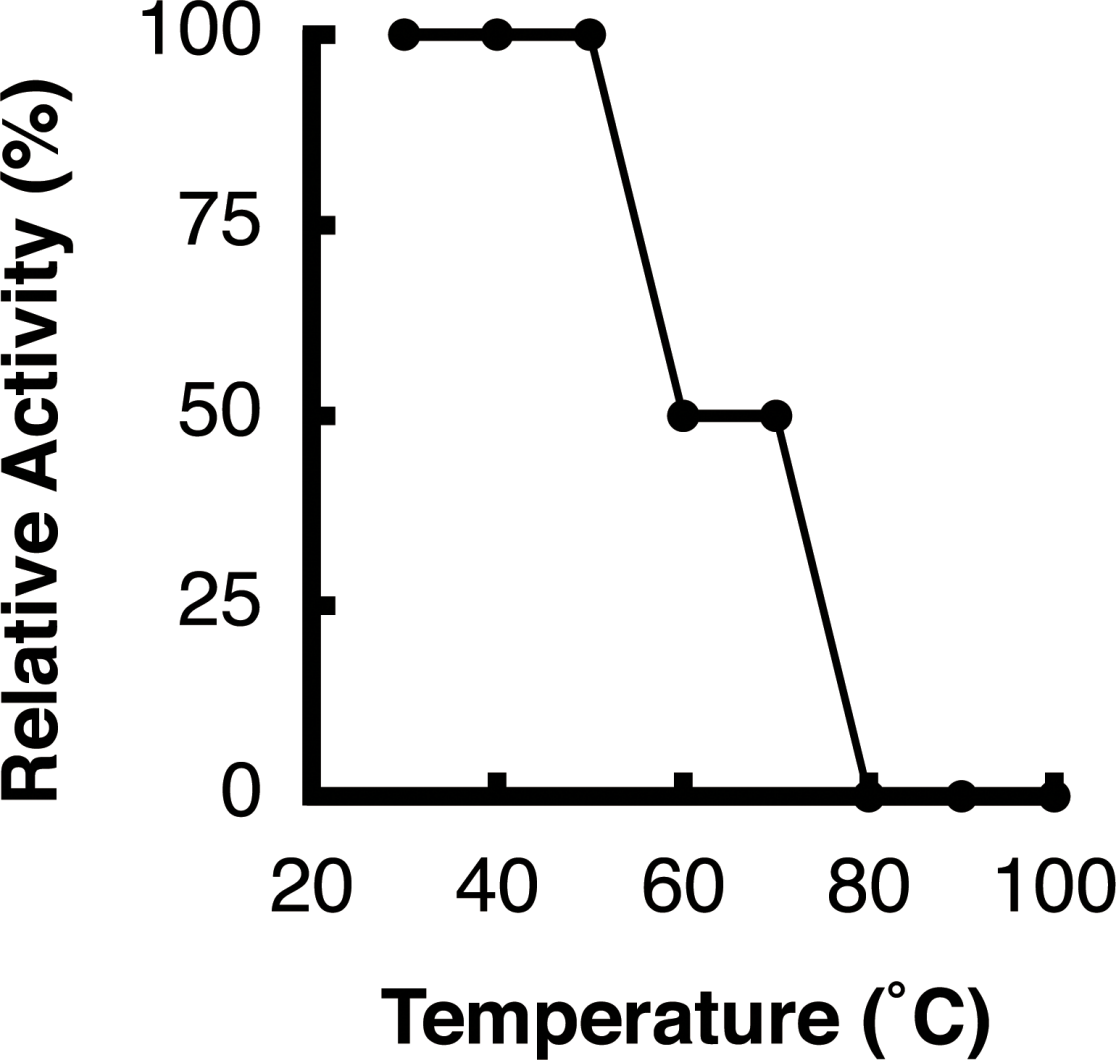
Thermal sensitivity of the antibacterial activity of the *P*. *subcostata* extract. The extract was incubated for 10 min at the indicated temperatures and subjected to an agar well diffusion assay. Relative antibacterial activity was calculated with activity from samples treated at 30°C as 100%. Representative data from three independent experiments with similar results are shown.

To examine thermal stability, we measured the antibacterial activity of the extract after incubation for 10 min at 30, 40, 50, 60, 70, 80, 90 or 100°C. The relative antibacterial activity was expressed as the percentage of the maximum value at 30°C. The antibacterial activity was completely lost at temperatures over 80°C and was reduced by 50% at 60°C (Fig 2). This result indicated that the substance(s) with antibacterial activity in *P*. *subcostata* extract was thermo-labile.

Next, we determined the approximate molecular mass of the substance(s) showing antibacterial activity. The extracts were passed through three filters with molecular weight cutoffs of 10, 50, and 100 kDa. Each retentate and permeate fraction obtained after ultrafiltration was subjected to an agar well diffusion assay of its antibacterial activity. The retentate fractions from the 10, 50, and 100 kDa molecular-weight-cutoff filters exhibited antibacterial activity, indicating that the antibacterial substance(s) in *P*. *subcostata* extract has a molecular weight > 100 kDa. To identify the molecular mass of the antibacterial substance(s), we subjected the *P*. *subcostata* extract to purification using column chromatography. We first carried out ammonium sulfate precipitation, followed by anion exchange chromatography and gel filtration (Table 2). Antibacterial activity was measured at each step and converted to kanamycin resistance values (see Materials and Methods). The antibacterial activity in the sample after anion exchange chromatography and gel filtration was 24060 μg/μg protein and 4254 μg/μg protein, respectively (Table 2). The activity after gel filtration was lower than that after anion exchange chromatography, possibly because *P*. *subcostata* contains several antibacterial substances, one of which was lost during gel filtration.

**Table 2:** Partial purification of antibacterial component(s) from *P*. *subcostata*.

Purification Steps	Total Protein	Total Activity	Specific Antibacterial Activity
	(μg)	(mg)*	(μg**/μg protein)
Crude Extract	1144	650	568
100% Ammonium Sulfate Precipitation	1562	444	284
DEAE Sepharose FF	100	2400	24060
Toyopearl HW 65 S	18	75	4254

* Total activity shows antibacterial activity converted to kanamycin value (see materials and methods).

** Specific antibacterial activity shows kanamycin value (μg) per 1 μg proteins.

### HOX was associated with antibacterial activity in *P*. *subcostata* extract

**Fig 3:**
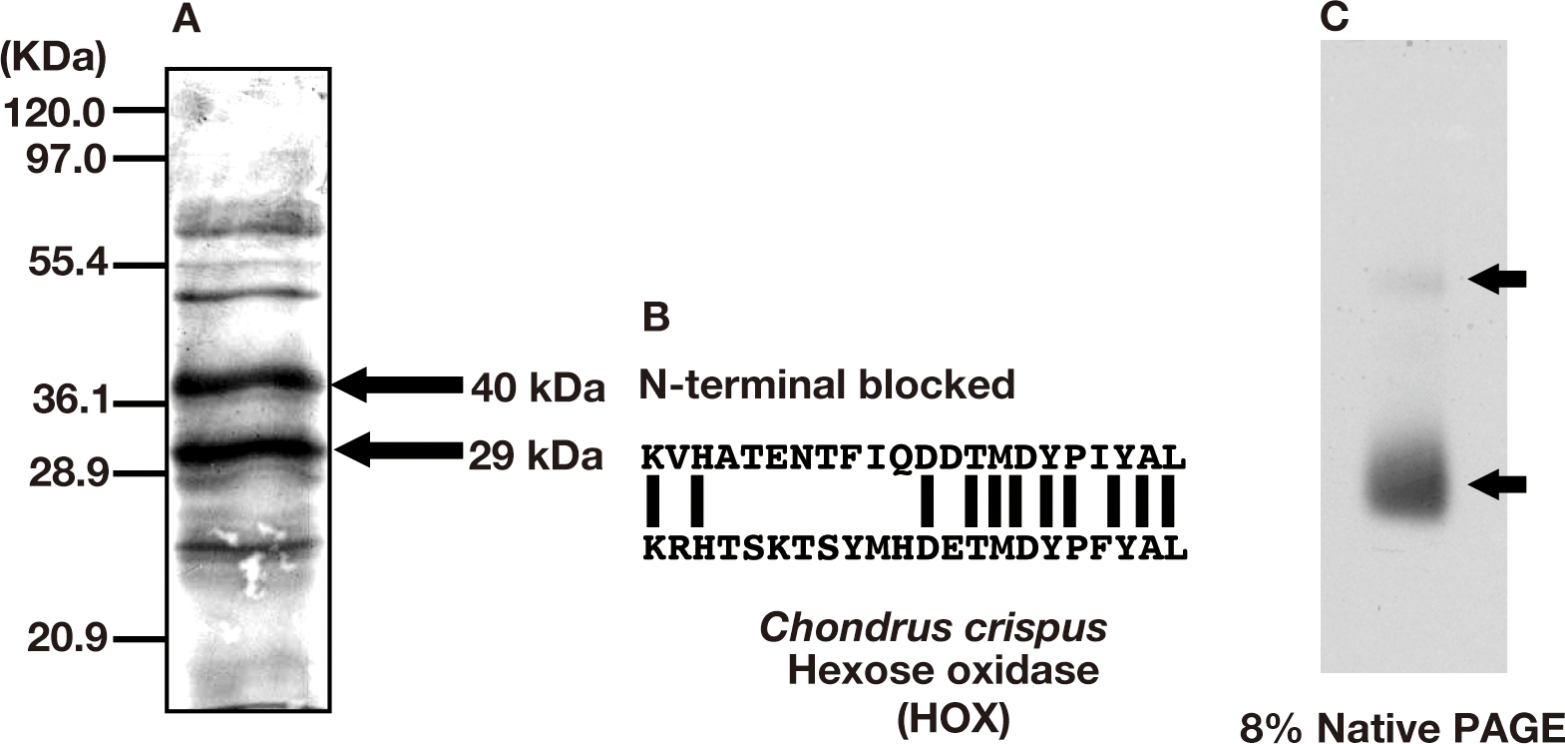
Association of HOX with the antibacterial activity of *P*. *subcostata*. (A) CBB staining image of the sample after SDS–PAGE. The bands indicated by arrows were subsequently subjected to N-terminal Edman degradation-peptide sequencing analysis. (B) Alignment of the N-terminal amino acid sequence of the 29 kDa polypeptide with that of *C*. *crispus* HOX. The amino acid sequence of the 29 kDa polypeptide shared 50% homology and 95.5% similarity with that of *C*. *crispus* HOX. (C) In-gel HOX activity assay. The sample was subjected to native PAGE and then stained with 4-chloro-1-naphthol to detect HOX activity. The arrows indicate the detected bands.

To identify the proteins with antibacterial activity, we subjected the partially purified sample to SDS–PAGE and then CBB staining. As shown in Fig 3A, two major bands with molecular masses of 29 kDa and 40 kDa were detected. We analyzed the N-terminal amino acid sequences of the 29 kDa and 40 kDa polypeptides using N-terminal Edman degradation. The N-terminal amino acid sequence of the 29 kDa polypeptide was determined to be VHATENTFIQDDTMDYPIYAL (Fig 3B). On the other hand, Edman degradation of the 40 kDa polypeptide failed to yield an identifiable amino acid, suggesting that the N-terminus is blocked. A BLAST database search showed that the N-terminal amino acid sequence of the 29 kDa polypeptide shared 50% identity and 95.5% similarity with a hexose oxidase (HOX) from the red alga *Chondrus crispus* [28] (Fig 3B). HOX can oxidize a variety of hexoses with concomitant reduction of molecular oxygen to H_2_O_2_ [29]. To examine HOX activity in the partially purified sample, we performed an in-gel H_2_O_2_ assay. The partially purified sample was separated by native-PAGE and then the gel was placed into solutions containing D-glucose and 4-chloro-1-naphthol (Fig 3C). In this assay, 4-chloro-1-naphthol is oxidized by H_2_O_2_ to generate a blue/purple compound. The two bands were detected specifically by this 4-chloro-1-naphthol staining (Fig 3C), suggesting that they correspond to the monomer and homodimer of HOX (see Discussion).

HOX has wide substrate specificity toward hexoses, whereas glucose oxidase (GOX) is highly specific for glucose [28]. To determine whether the partially purified sample was a HOX or GOX, we measured H_2_O_2_ generation in the presence of hexoses (D-glucose and D-galactose) and hexose disaccharides (lactose, cellobiose, and maltose) using a ferrous oxidation-xylenol orange (FOX) assay. The partially purified sample produced H_2_O_2_ from all of these hexoses (Table 3). This result suggested that these hexoses were substrates of the isolated enzyme and that the enzyme was HOX, rather than GOX.

**Table 3:** Substrate specificity for *P*. *subcostata* HOX.

Sugar	H_2_O_2_
	(μmol H_2_O_2_ / μg protein)
D-Glucose	156
Lactose	152
Cellobiose	147
D-Galactose	190
Maltose	158

### The antibacterial activity of *P*. *subcostata* was mediated by H_2_O_2_ produced by HOX

To determine whether the H_2_O_2_ associated with the identified HOX mediates the antibacterial activity of *P*. *subcostata*, we first investigated the effect of catalase on the bacterial growth-inhibitory activity in algal extract. We incubated the *P*. *subcostata* extract in the presence or absence of catalase for 16 h and subsequently examined its effect on bacterial growth. The treatment of 1 mg/ml and 2 mg/ml catalase completely abolished the antibacterial activity of the *P*. *subcostata* extract (data not shown).

**Fig 4:**
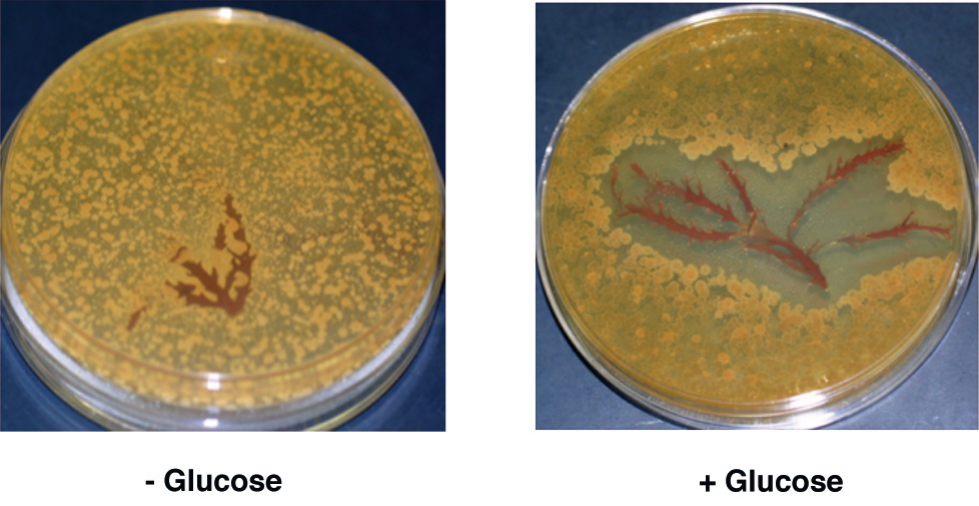
Suppression of *B*. *subtilis* growth near *P*. *subcostata*. Algal fronds of *P*. *subcostata* were placed on a GYP agar plate onto which *B*. *subtilis* spores had been spread, then incubated at 37°C for 18 h. Bacterial colony formation was strongly suppressed near *P*. *subcostata* algal fronds.

Next, to examine whether the HOX from *P*. *subcostata* required hexose for its antibacterial activity, we placed algal fronds on GYP agar plates with or without D-glucose inoculated with *B*. *subtilis* spores. *B*. *subtilis* spores grew on the GYP agar plates without D-glucose in the presence of algal fronds. In contrast, bacterial growth was strongly suppressed near the algal fronds on GYP agar plates containing D-glucose (Fig 4).

## Discussion

### Red algae, *P*. *subcostata* and *C*. *crispus*, HOX might form heterodimar

The form of an enzyme is and important determinant of its activity. Our results suggested that *P*. *subcostata* HOX might form a homodimeric structure, as did the HOX of *C*. *crispus*. The molecular mass of *C*. *crispus* HOX was reported to be approximately 110 kDa [28] and 130 kDa [30]. Hansen and Stougaard have suggested that the open reading frame of the isolated cDNA corresponded to a polypeptide of molecular mass 62 kDa, and that the enzyme formed a homodimeric structure [28]. Hansen and Stougaard [28] also reported that the purified *C*. *crispus* HOX migrated as a single band in native PAGE, where three bands of molecular mass 29 kDa, 40 kDa, and 62 kDa were observed in SDS-PAGE and these molecular masses suggested that the two smaller polypeptides were cleavage products derived from the 62 kDa polypeptide. Similarly, in *P*. *sbucostata* HOX, two bands of molecular mass 29 kDa and 40 kDa in SDS-PAGE might be cleavage products derived from the polypeptide and it might formed a homodimeric structure. The antibacterial activity in *P*. *subcostata* extract was suggested to be mediated by HOX, a homolog of the *C*. *crispus* HOX (Fig 3). *C*. *crispus* HOX is synthesized as a precursor propeptide and then cleaved into two smaller polypeptides determined by SDS-PAGE to have molecular masses of 40 kDa and 29 kDa [28]. The data in Fig 3A suggest that the 40 kDa and 29 kDa polypeptides of *P*. *subcostata* were HOX. These polypeptides might be produced by cleavage from the corresponding precursor propeptide of HOX, similar to *C*. *crispus* HOX. Thus, HOX might be conserved between these two species of red algae. More determination of the amino acid sequences of these two bands from *P*. *subcostata* would support the conclusion that the two bands are indeed corresponding to HOX and it was responsible for the antibacterial activity.

### Possible significance of HOX-mediated H_2_O_2_ production in *P*. *subcostata*

HOX has the highest substrate specificity for hexoses. *C*. *crispus* HOX has broad substrate specificity for hexoses [28, 30, 31]. It was exhibited by the enzyme with respect to hexoses (D-glucose and D-galactose) and disaccharides (lactose, cellobiose and maltose) and the activity ratios were high with hexoses and low with disaccharides [28, 30, 31]. On the other hand, our results suggested that the substrate specificity of the *P*. *subcostata* HOX did not differ markedly between hexoses and disaccharides unlike the *C*. *crispus* HOX. What is the natural substrate of HOX in *P*. *subcostata*? One candidate is galactose, which is a major component of the cell walls of marine red algae [14, 16]. Interestingly, H_2_O_2_ was produced most abundantly in the presence of galactose (Table 3). Galactose derived from cell-wall injury might act as a substrate for HOX.

*C*. *crispus* HOX was suggested to be synthesized as a precursor prepropeptide that contains a signal peptide for secretion and is presumably localized on the cell surface [28]. Similarly, *P*. *subcostata* HOX might be present on the cell surface. What is the biological significance of the cell surface localization of HOX and H_2_O_2_ production? There are at least two hypotheses, both of which involve algal immunity. The first hypothesis is direct activity against microbes. H_2_O_2_ production strengthens algal defense at the site of injury to prevent microbial invasion of the algal tissue. The second hypothesis is an indirect role of H_2_O_2_ on algal antimicrobial defenses. H_2_O_2_ might be responsible for oxidative cross-linking of cell wall components to protect the alga [14, 32, 33]. HOX-produced H_2_O_2_ might be involved in cell wall repair in association with antibacterial activity. Purification of the HOX enzyme in a further study would enable its role in H_2_O_2_ production and antibacterial activity to be determined.

### Differences in substrate specificity between HOX and GOX may reflect differences in the defense systems of marine algae and terrestrial plants

Honeydew from terrestrial plant flowers has antibacterial activity [27, 34]. Tobacco nectarin V is a flavin-containing berberine bridge enzyme-like protein with GOX activity that is involved in the defense against bacterial infection in nectar [27]. Nectarin V is secreted into nectar in flowers and metabolizes the glucose in nectar to generate H_2_O_2_ [27]. The production of high levels of H_2_O_2_ restricts the growth of microorganisms, which are spread by insects as they suck nectar [27]. It is interesting to note that terrestrial plants contain GOX in nectar, while seaweeds contain HOX in their fronds. We suspect that these differences arise from differences in their polysaccharide composition. The disaccharide sucrose and its component glucose are major sugars in nectar [35], while various polysaccharides exist in the cell walls and intercellular spaces of algal fronds [16]. *P*. *subcostata* extract had the ability to resist a broad spectrum of bacteria (Table 1). Pathogenic bacteria to seaweeds tend to be opportunistic [2]. However, it remains unclear whether HOX-mediated H_2_O_2_ production is a functional mechanism to resist pathogenic bacteria in marine environments. Therefore, further studies are required to make sure that whether HOX contribute to the red algae innate immune system or not. Comparison of the defense systems of seaweeds and terrestrial plants will increase our understanding of the evolution of plant immunity in different environments.

## Supporting Information

S1 Fig Antibacterial activity of kanamycin. (A) 96 well plate assay showing growth inhibition of *B*. *subtilis* by kanamycin treatment. Upper 4 wells, treated with kanamycin 0.0125 μg / well; Lower 4 wells, treated with kanamycin 0.025 μg / well. Growth-inhibition was observed in lower 4 wells. (B and C) Microscopic image of the solution of upper 4 wells (B) and lower 4 wells (C). *B*. *subutilis* was proliferated in (B), but not in (C). (D) *B*. *subutilis* was cultured with or without kanamycin in 96 well titer plates and measured OD 660 nm.
(PDF)
