# Supplementary material for: Hexose Oxidase-Mediated Hydrogen Peroxide as a Mechanism for the Antibacterial Activity in the Red Seaweed Ptilophora subcostata
Source: PLoS One. 2016 Feb 11;11(2):e0149084. doi: 10.1371/journal.pone.0149084 (PMC4750953; doi:10.1371/journal.pone.0149084)
Supplement: S1 Fig — (A) 96 well plate assay showing growth inhibition of B. subtilis by kanamycin treatment. Upper 4 wells, treated with kanamycin 0.0125 μg / well; Lower 4 wells, treated with kanamycin 0.025 μg / well. Growth-inhibition was observed in lower 4 wells. (B and C) Microscopic image of the solution of upper 4 wells (B) and lower 4 wells (C). B. subutilis was proliferated in (B), but not in (C). (D) B. subutilis was cultured with or without kanamycin in 96 well titer plates and measured OD 660 nm. (PDF) [file pone.0149084.s001.pdf]

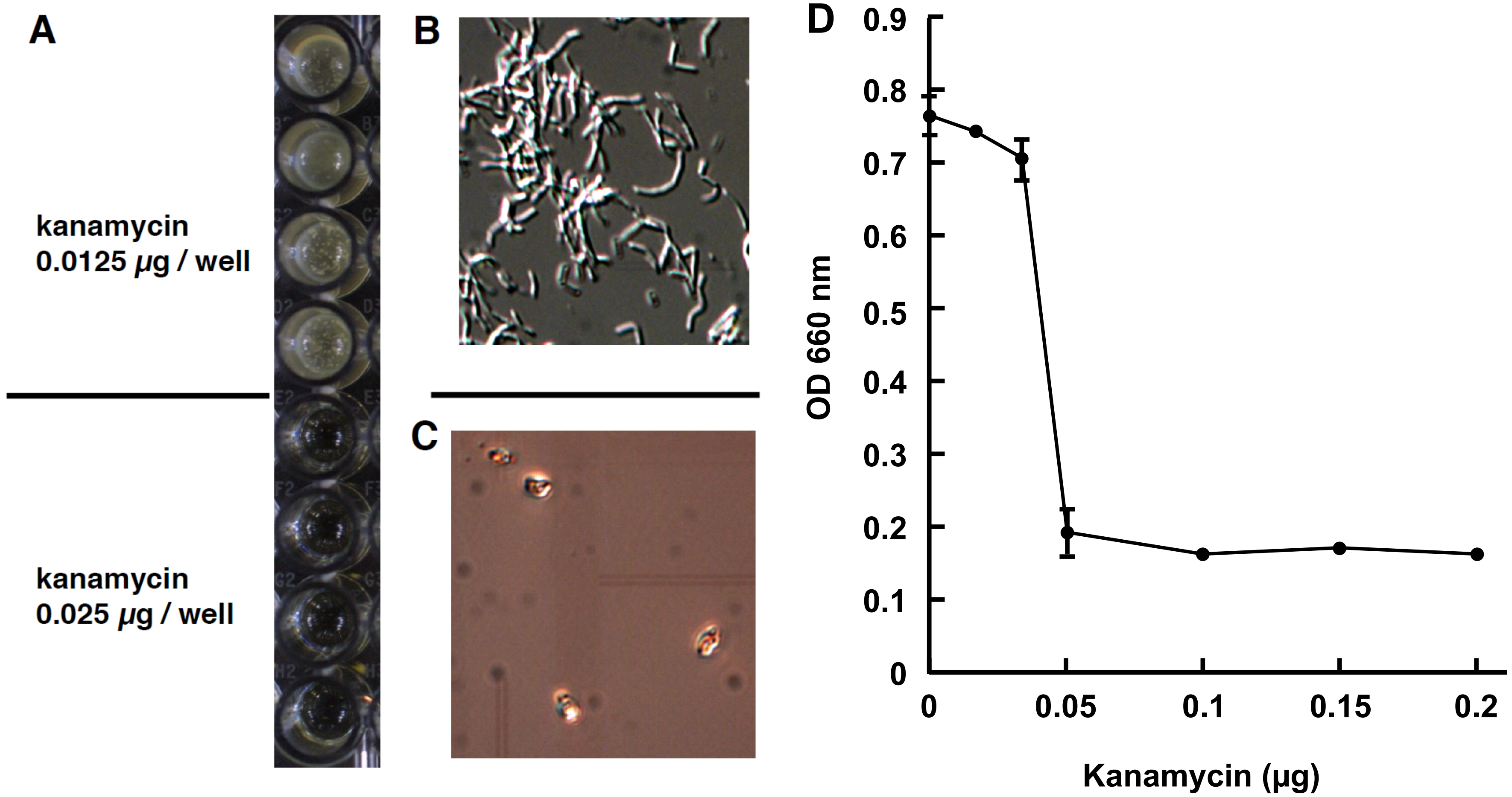

Supplemental Fig.1. Antibacterial activity of kanamycin. (A) 96 well plate assay showing growth inhibition of *B. subtilis* by kanamycin treatment. Upper 4 wells, treated with kanamycin 0.0125 μg / well; Lower 4 wells, treated with kanamycin 0.025 μg / well. Growth-inhibition was observed in lower 4 wells. (B and C) Microscopic image of the solution of upper 4 wells (B) and lower 4 wells (C). *B.subutilis* was proliferated in (B), but not in (C). (D) *B.subutilis* was cultured with or without kanamycin in 96 well titer plates and measured OD 660 nm.
